# Spotlight on early-career researchers: an interview with Ragothaman Yennamalli

**DOI:** 10.1038/s42003-018-0146-z

**Published:** 2018-10-10

## Abstract

Ragothaman Yennamalli is an Assistant Professor at Jaypee University of Information Technology in Waknaghat, India, where he uses computational tools to study protein structure and function. In this installment of our Q&A series with early-career researchers, Ragothaman tells us about his journey from microbiology to computational biology and the inspiration and challenges he experienced along the way.

**Figure:**
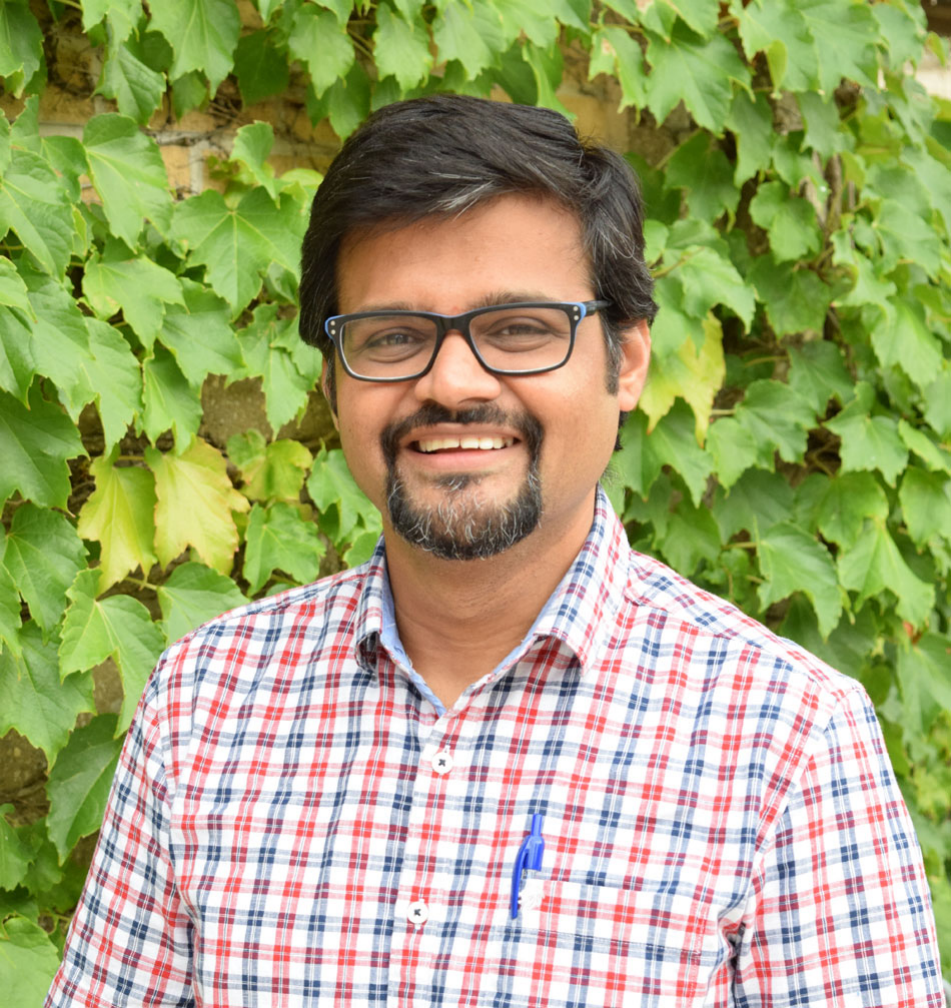
Image credit: Ragothaman Yennamalli

Please tell us about your research interests.

My research interests are broadly in the area of computational biology and bioinformatics. More specifically, in enzymes that catalyze interesting reactions and their interplay with other proteins. Currently, my focus is on lytic polysaccharide monooxygenases that are important for bioethanol production. These proteins are not only industrially important but also play a role in bacterial pathogenesis during invasion of the host. Initially, we are looking at the protein structure and dynamics to understand their interaction with crystalline polysaccharide substrates like Cellulose and Chitin, as it is crucial to look at both static and dynamic properties of proteins to get the big picture.

What has your journey been to this point?

My journey started with my love for biology in high school, where the seeds were planted by Mrs. Kamalam Nair (High school biology teacher) and Mr. Subramani (high school physics teacher) who taught me to look at science and the scientific process not just for getting grades, but for fun! Thus, my journey has always gravitated towards doing science that makes me happy and is fun. I wanted to be a surgeon and prepared for years, but ended up loving microbes and studied Microbiology as an undergrad. I was trained in wet lab science, but I transitioned to bioinformatics after my masters as I found it equally appealing and exciting. I loved to code and use computers to ask the questions. At every life turning step, I was fortunate to have the support and guidance of my mentors who helped me make tough decisions. Thus, I ventured into my doctoral research with Dr. Subbarao at Jawaharlal Nehru University, as the other option of working a typical “nine-to-five” job was not fun and didn’t tickle my grey matter. Thanks to the Australian government, I visited the labs of Prof. Bostjan Kobe, Prof. Alan Mark, and Prof. Paul Young during my PhD. They taught me cell culture along with computational simulations. It was after earning my PhD that I wanted to move away from drug design and jumped into the unfamiliar world of bioenergy.

Realizing that I was a novice in bioenergy, Dr. Taner Sen and Prof. Jeff Wolt at Iowa State University kept me engaged and motivated with their discussions on my project. I went out of my comfort zone to learn new things and expand my horizons. I think this thinking led me to work with Prof. George Phillips (UW-Madison and then at Rice University). During my postdoc I learned that some skills were waiting to be discovered and are basically inherent with us. Specifically, I found that I was good at managing a team and being a team-player. It was fun working with George as we ended up setting an 80 inch HDTV linked to Xbox controllers for visualizing protein structures in 3D. It was a mini home theatre setup in the lab!

Experiences at every step of my life has motivated me to be a better person and a better scientist. I have tried to apply these skills in my independent position at JUIT, where I have been for the nearly three years. At the same time, I am trying to have fun along the way.

What are your predictions for your field in the near future?

The Computational Biology and Bioinformatics fields are at a very exciting stage right now. Lots of hard working and inspiring scientists are making the field interdisciplinary and cross disciplinary. The huge amount of data getting churned out every day poses challenges to analyze the data, requiring many labs and personnel. Specifically, I foresee high-throughput data driven quantifiable outcomes that use machine learning for applications ranging from personal genomics to molecular systems. Also, data sharing and access will drive the future of my field. This is exciting to me as the data will enable new advances and new opportunities that will lead to new perspectives.

Can you speak of any challenges that you have overcome?

I think a major challenge for me has been imposter syndrome. I didn’t know there was a specific term for it until I read about it and I think a light bulb switched on! I better understood how to tackle this syndrome and not to give in to it during my crucial moments of research life. Another challenge is to keep myself positive and motivated. I accepted the fact that one has to draw energy from small victories and like Dory says in the Disney film *Finding Nemo*, “Just keep swimming” works like a charm. I have to confess here that my late parents’ advice “all for good” has helped me see the best in adverse situations.

As a new PI, I face challenges of time-management and the fear of not being a good mentor for my students. I also face the challenge of expectations. With time, I think, I am getting better at this. I also have to say that for the last year or so, the NewPI Slack community (~1000 members) consisting of Assistant Professors has helped me get to where I am now. It has been a godsend to get help and support from peers who are excellent scientists, and an amazing soundboard for bouncing ideas.

What advice would you give to your younger self?

First, be kind to yourself. Don’t’ beat yourself up! Things may not look like it is going to be ok, but they turn out alright.

Second, always rely upon your gut instincts and make sure to have fun!

Third, get into networking as early as possible. Some of my most fruitful collaborations have been from contacts I made many years ago.

Bonus question: What is your favorite protein structure?

That’s a tough question to answer! There are lots of proteins I have worked with and each one of them is unique and intriguing. However, there is one that probably made me take a plunge into protein structure and function: the structure of Bovine Ribonuclease Inhibitor. I wrote an assignment and gave a presentation to my peers about its function back in 2001. I was drawn to it because it looked like a horseshoe, and indeed it belongs to the horseshoe fold of proteins. Three years later when I worked with Prof. Bostjan Kobe at the University of Queensland in Australia, I realized that solving this protein structure was his postdoctoral work with the Nobel Laureate Prof. Johann Deisenhofer. I had no clue at all when I started and I was amazed at the way things took a turn. So, that protein left an indelible memory.

*This interview was conducted by Chief Editor Brooke LaFlamme*

